# Effects of recombinant human growth hormone administration on cardiovascular risk factors in obese children with relative growth hormone deficiency

**DOI:** 10.1186/s12944-018-0721-9

**Published:** 2018-04-03

**Authors:** Shuang Liang, Jiang Xue, Guimei Li

**Affiliations:** 1 Department of Pediatrics, The Second Hospital of Shandong University, 247 Beiyuan Main Street, Jinan, 250021 Shandong China; 2 Department of Pediatrics, Shandong Provincial Hospital Affiliated to Shandong University, 9677 Jingshi Road, Jinan, 250021 Shandong China

**Keywords:** Recombinant human growth hormone, Relative growth hormone deficiency, Obese children

## Abstract

**Background:**

Based on the sample of obese children with relative growth hormone deficiency (GHD), the objective of our study was to determine the effects of rhGH treatment on cardiovascular risk factors, including body mass index (BMI), lipid levels and glucose metabolism index.

**Methods:**

A total of 43 obese children with relative GHD were included in our final analysis. The obese subjects were divided into two groups: recombinant human growth hormone (rhGH) treatment group and untreated control group.

**Results:**

After 6 months, subjects in the rhGH treatment group had significant reductions in BMI standard deviation scores (SDS) compared with controls (2.32 ± 0.85 vs. 2.80 ± 0.61; *P* = 0.041), and Insulin-like growth factor 1(IGF-1) level increased during rhGH treatment, in comparison with the control group (702.91 ± 246.03 vs. 348.30 ± 131.93 ng/mL, *P* < 0.001). GH treatment reduced low density lipoprotein cholesterol (LDL-C) (2.20 ± 0.45 vs. 2.63 ± 0.76 mmol/L, *P* = 0.027), aspartate aminotransferase (AST) (21.26 ± 5.72 vs. 32.30 ± 17.68 mmol/L, *P* = 0.006) as well as alanine aminotransferase (ALT) (16.70 ± 6.72 vs. 45.20 ± 46.62 mmol/L, *P* = 0.002), and increased high density lipoprotein cholesterol (HDL-C) (1.45 ± 0.40 vs. 1.19 ± 0.23 mmol/L, *P* = 0.016) levels compared with the control group.

**Conclusion:**

RhGH treatment for 6 months on obese children with relative GHD reduces BMI SDS, stabilize IGF-1 levels, and exerts beneficial effects on blood lipid profiles and live enzyme compared with untreated control group. Moreover, GH administration has no significant effects on increased insulin resistance and no adversely effect on glucose homeostasis.

## Background

Due to the increasing incidence rate for children and teenagers, obesity has gradually become a global social problem. Obesity has always been linked with insulin resistance, hypertension, dyslipidaemia, hyperglycemia and cardiovascular disease, then adversely impacts the children’s long-term health. Early intervention should be the effective strategies to avoid the complications of obesity. Life style modification and caloric restriction are the common treatment options for childhood obesity treatment. However, it has a same limitation which is the low success rate. A recent study conducted by Scicchitano et al. [1] provided some evidences relating to the positive effects of nutraceuticals and functional food action on dyslipidaemia and cardiovascular risk factors. Although the main mechanisms of their positive effects on the cardiovascular system was still not clear in the above study, it showed that nutraceuticals and functional food could reduce the cardiovascular related complications, and it presented a possible research direction for a forthcoming therapeutic approach for obesity-related metabolic and cardiovascular complications. Except for the above methods, other possible methods like pharmacological treatment have not been widely utilized.

Apart from the common obesity-related co-morbidities, obesity is also associated with the abnormalities in the growth hormone (GH)- insulin-like growth factor 1 (IGF-1) axis. It is mainly reflected in that, for both children and adults, obesity may cause the impaired spontaneous GH secretion [2, 3] and the decreased GH response to stimulation test [4, 5]. The obesity-related GH deficiency (GHD) is reversible with weight loss, and thus was always treated as relative and functional. During the last decades, several studies started to focus on the research questions around the association between functional hyposomatotropism and cardiometabolic risk markers in obesity. In 2008, Utz et al. [6] firstly proposed the concept of relative GHD syndrome in overweight and obese women without organic pituitary or hypothalamic disease, and found the inverse associations between the relative GHD syndrome and cardiovascular risk markers. Makimura et al. [7] showed that, for obese adults, there were strong inverse associations between GH levels and carotid intima-media thickness (cIMT), which represents the early indicators of atherosclerosis. In addition, other studies have also confirmed that a negative association also exsits between GH secretion and cardiovascular risk factors in obese patients with normal pituitary function [8–10].

Collectively, all the above studies suggested that the reduced GH secretion in obesity might cause cardiovascular consequences. Thus we assume that recombinant human growth hormone (rhGH) treatment can be used to improve obesity-related cardiovascular metabolic complications. Previous studies have shown that rhGH treatment for obese adults, who exhibit similar GH axis abnormalities as obese children, can reduces abdominal obesity and improves insulin sensitivity, as well as blood lipid profiles [11–14]. However, few of them have investigated the association between cardiovascular risk factors and relative GHD in obese children. Moreover, whether rhGH can improve complications of childhood obesity in obese with relative GHD children have not been studied, too. Due to the importance of such research questions we mentioned before, relative studies are very necessary to further confirm the effects of rhGH as a treatment on obese children with relative GHD.

In order to fill the above research gaps, this study conducted a set of experiments by requesting the obese participants with relative GHD to receive a 6 month of rhGH treatment. The objective of this study is to assess the effect of rhGH treatment on cardiovascular risk factors, including BMI, lipid levels, insulin resistance index and glucose metabolism index in obese patients with relative GHD. This study also intends to assess the safety in treating obese children with relative GHD. Based on the completion of the above purposes, we attempt to confirm whether rhGH treatment can be treated as a potential treatment for obese children with relative GHD.

## Methods

The study was approved by the Ethics Committee of the Second Hospital of Shandong University and Shandong Provincial Hospital Affiliated to Shandong University, which has the written informed consent from all and all subjects’ parents.

### Subjects

Subjects were recruited from the Department of Pediatric Endocrinology of the Second Hospital of Shandong University and Shandong Provincial Hospital Affiliated to Shandong University between July 2014 and July 2017. The diagnosis of obese children with relative GHD was obese subjects, which meet standard of diagnostic criteria used to diagnose GHD in hypopituitarism but without organic pituitary or hypothalamic disease. The criteria for inclusion in the study were: the body mass index (BMI) of children aged from 8 to 18 years should be higher than 95 percentile for normal weight children of the same age and gender [15]. The standard of GH stimulation test (arginine test and levodopa test) is: GH peak < 10 μg/L. Exclusion criteria included: 1) hypothalamic or pituitary disorders, hypothyroidism, adrenal dysfunction; 2) diabetes mellitus, chromosome abnormalities or all sorts of syndromes; 3) hepatitis B or C, serious infection, systemic disease and other chronic wasting illnesses; 4) the use of drugs may influence body composition, GH secretion or glucose and lipid metabolism; 5) short stature or the growth velocity is less than 5 cm/year; 6) highly allergic to all or susceptible to the test drug.

**Fig. 1:**
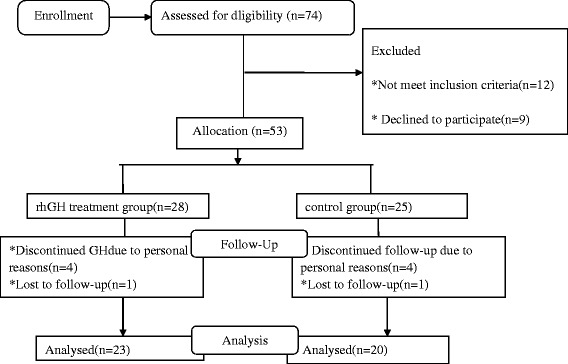
Flow chart of the study

A total of 53 children met the criteria and were selected for participation in the study. Eight subjects withdrew for personal reasons and two subjects were lost to follow-up. Finally, 43 subjects completed the 6-month visit (Fig. 1).

The obese children were then divided into two groups: a test group consisting of 23 subjects, who opted to receive the rhGH treatment, and a control group consisting of 20 subjects. Children in the control group opted not to receive the rhGH treatment but agreed to a regular follow-up. No diet and exercise modifications were initiated of all subjects.

Anthropometric measurements, laboratory examinations, and imaging parameters were performed on all children. The following parameters were collected at baseline: height, weight, pubertal stages, GH stimulating tests, free triiodothyronine (FT3), free thyroxine (FT4), thyroid-stimulating hormone (TSH), adrenal corticotropic hormone (ACTH), cortisol (COR), IGF-1, total cholesterol (TC), high density lipoprotein cholesterol (HDL-C), low density lipoprotein cholesterol (LDL-C), triglycerides (TG), alanine aminotransferase (ALT), aspartate aminotransferase (AST), fasting blood glucose (FBG), fasting insulin, hypothalamic-pituitary magnetic resonance imaging (MRI) and bone age (BA) were performed in all children. After baseline evaluation, rhGH treatment group was to receive rhGH replacement therapy. The rhGH dose was 0.23–0.35 mg/kg/week (Changchun JinLei SaiZeng Co.), once daily subcutaneous in every night before bedtime, and the rhGH dose was read just according to weight every 3 month. Anthropometric measurements, thyroid function, IGF-1, liver function, FBG, lipid profile, glycosylated haemoglobin (HbA1C), hemogram, urinalysis, and urinary calcium were reassessed after 3 month and 6 month in rhGH treatment group and control group.

### Auxological measurements

Standing height was measured to the nearest 0.1 cm using a stadiometer. Weight was weighed to the nearest 0.1 kg on a standard electronic scale, with the subject dressed light clothing without shoes. BMI was calculated as the ratio between body weight in kilograms and height in meters squared. To minimize the confounding effects of age and sex, BMI SDS (standard deviation scores) were calculated using reference values in Chinese children [16]. The pubertal developmental stage was assessed by physical examinations, according to the criteria established by Tanner [17].

### Laboratory examinations

All laboratory measurements were made in the morning after a 12 h fast using standardized methods. GH secretion was evaluated using two GH stimulation test: arginine test (0.5 g/kg, maximum 30 g) and levodopa test (10 mg/kg, maximum 0.5 g). Serum GH levels were determined at 0, 30, 60,90, 120, 150 min after two stimulation tests. GH peak< 10 μg/L was considered as relative GHD. Serum GH level was measured using chemiluminescence assay (Cobas E170, Roche Diagnostics, Germany). Serum FT3, FT4, TSH, ACTH, COR and IGF-1 were measured using chemiluminescence assay (Siemens Healthcare Diagnostics, USA). TC, HDL-C, LDL-C, TG, FBG, ALT and AST were measured using an Auto Biochemical Analyzer (AU5400, Beckman Coulter, Tokyo, Japan). Fasting insulin were measured using a chemiluminescent immunometric assay (CobasE170, Roche Diagnostics, Mannheim, Germany). The intra-assay and inter-assay coefficients of variation were all < 8.0% in these assays. Insulin resistance was estimated using the homeostasis model assessment for insulin resistance (HOMA-IR) as follows: insulin (uIU/mL) × glucose (mmol/L)/22.5 [18]. An oral glucose tolerance test (OGTT) was performed with sampling for plasma glucose ≥5.6 mmol/L subjects in order to rule out diabetes mellitus. The assessment of HBsAg and antibody titer for HCV was performed in subjects with abnormal liver function (ALT> 50 U/L) in order to rule out viral hepatitis.

### Imaging parameters

All obese children underwent hypothalamic pituitary MRI examination. MRI scans was performed using a 3.0 T Scanner (Siemens, Erlangen, Germany) in the sagittal and coronal planes on T1 weighted imaging and T2 weighted imaging with 3 mm thickness. The morphology of the pituitary, the height of the anterior pituitary, the position and size of the posterior pituitary, the shape of the pituitary stalk, and other abnormal manifestations of the sellar region and the sellar structures were observed.

BA was determined by radiograph of the left hand and wrist according to the method of Greulich and Pyle. Then we calculated the bone age-chronological age (BA–CA).

All participants with liver damage were examined by liver ultrasonography which was performed in all subjects with abnormal liver function. Ultrasonic scanning of the liver was performed using a 3.5–5 MHz Voluson E8 transducer (GE Healthcare, Tiefenbach, Austria) after subjects had observed a 12-h fast.

## Statistical analysis

Parametric variables were expressed as the mean (standard deviation). Nonparametric variables were log transformed before analysis and were expressed as the mean (standard deviation), and the variables were expressed as median (interquartile range) since they cannot be transformed to normal distribution. Two independent-sample t test and Mann-Whitney test were used for comparing differences between rhGH treatment subjects and untreated group. Categorical variables were compared by chi square test. Multivariate regression models were constructed to inviestigate whether the changes in endpoints persisted after controlling for baseline age, gender and IGF-1 level are significant. A *P* value of < 0.05 was considered statistic significance. Statistical analyses were performed by SPSS version 20.0 (SPSS Inc. Chicago, USA).

## Results

### Baseline characteristics

Baseline characteristics of all subjects are described in Table 1. Twenty-three subjects were opted to receive rhGH treatment and twenty subjects do not receive the treatment of rhGH. In rhGH treatment group, with the mean age of 11.61 ± 1.73 years old, the subjects include 21 boys and 2 girls. It shows that most of the participants were male (91.3%), and the majority of children were prepubertal (16, 69.6%) with an average GH peak of 1.8 (1.20-3.70)μg/L. In the control group which consists of 19 boys and 1 girl, the average age was 11.25 ± 1.86 years old. Fourteen of 20 subjects were prepubertal (70%), and the mean GH peak is 1.75 (1.23-3.35) μg/L. In addition, in these two groups, impaired liver function was observed in 6 of 23 in rhGH treatment group participants (26%) and 6 of 20 (30%)in the control group. The obese children with impaired liver function all underwent liver ultrasonography, were all diagnosed with nonalcoholic fatty liver disease (NAFLD).

**Table 1:** Baseline characteristics of all obese with relative GHD children

Variable	rhGH group (*n* = 23)	Control group (*n* = 20)	*P* value
Age (yr)	11.61(1.73)	11.25(1.86)	0.516
Male/Female (*n*)	21/2	19/1	0.635
Prepuberty/Puberty, (*n*)	16/7	14/6	0.975
BMI SDS	2.64(0.74)	2.73(0.71)	0.676
BA-CA(yr)	1.43(1.88)	1.32(1.63)	0.826
Peak stimulated GH(μg/L)	1.8(1.2-3.7)	1.75(1.23-3.35)	0.761▲
IGF-1(ng/ml)	241.47(105.98)	291.1(151.95)	0.321#
TC(mmol/L)	4.81(0.65)	4.34(1.05)	0.093
HDL-C (mmol/L)	1.32(0.39)	1.22(0.22)	0.405#
LDL-C (mmol/L)	2.87(0.47)	2.58(0.85)	0.084#
TG(mmol/L)	1.49(0.76)	1.22(0.38)	0.326#
ALT(U/L)	22(15-64)	23(16.25-68.75)	0.567▲
AST(U/L)	25(22-32)	26.5(20.25-46.25)	1▲
Insulin(uIU/mL)	28.32(17.23)	27.92(16.73)	0.807#
FBG(mmol/L)	5.21(0.43)	5.12(0.43)	0.527▲
HOMA-IR	6.63(4.35)	6.38(3.92)	0.746#
HbA1c (%)	5.7(5.4-5.9)	5,6(5.325-5.875)	0.448

*BMI SDS* body mass index standard deviation scores, *BA–CA* Bone age –chronological age, *GH* growth hormone, *IGF-1* Insulin-like growth factor 1, *TC* total cholesterol, *HDL-C* High density lipoprotein cholesterol, *LDL-C* Low density lipoprotein cholesterol, *TG* triglycerides, *ALT* alanine aminotransferase, *AST* aspartate aminotransferase, *FBG* fasting blood glucose, *HOMA-IR* homeostasis model assessment of IR, *HbA1c* Glycosylated haemoglobin

#*P* value reported for log transformed values, but values in the table represent a back transformation to the original

▲Mann-Whitney U test or chi square test

There were no significant differences in age, gender and pubertal stages for the above two groups. All the subjects had accelerated bone age (higher BA-CA 1.43 ± 1.88 vs. 1.32 ± 1.63; *P* = 0.826). At baseline, the two groups were comparable in BMI SDS, IGF-1 level, TC, HDL-C, LDL-C, TG, ALT, AST, HOMA-IR, insulin and FBG.

### Change in variables after 3 month and 6 month of rhGH treatment

Clinical and endocrine metabolic changes in obese with relative GHD children in rhGH treatment group and control group after3 month and 6 month are summarized in Tables 2 and 3.

**Table 2:** Change in variables in obese with relative GHD children treated with GH vs control group for 3 months

Variable	rhGH group (*n* = 23)	Control group (*n* = 20)	*P* value
BMI SDS	2.42(0.77)	2.68(0.53)	0.211
IGF-1(ng/ml)	587.35(271.74)	345.6(131.75)	0.001*
TC(mmol/L)	4.07(0.63)	4.23(0.78)	0.457
HDL-C (mmol/L)	1.45(0.38)	1.17(0.24)	0.005*
LDL-C (mmol/L)	2.28(0.48)	2.54(0.55)	0.107
TG(mmol/L)	1.11(0.50)	1.15(0.32)	0.762#
ALT(U/L)	25.33(17.19)	49.50(55.91)	0.074#
AST(U/L)	26.2(8.13)	32.4(21.8)	0.427#
Insulin(uIU/mL)	34.35(20.06)	29.52(28.41)	0.177#
FBG(mmol/L)	5.39(0.37)	5.19(0.35)	0.073
HOMA-IR	8.26(5.04)	7.03(7.15)	0.14#
HbA1c (%)	5.63(0.37)	5.63(0.39)	0.959#

*BMI SDS* body mass index standard deviation scores, *IGF-1* Insulin-like growth factor 1, *TC* total cholesterol, *HDL-C* High density lipoprotein cholesterol, *LDL-C* Low density lipoprotein cholesterol, *TG* triglycerides, *ALT* alanine aminotransferase, *AST* aspartate aminotransferase, *FBG* fasting blood glucose, *HOMA-IR* homeostasis model assessment of IR, *HbA1c* Glycosylated haemoglobin

*P<0.05

#*P* value reported for log transformed values, but values in the table represent a back transformation to the original

**Table 3:** Change in variables in obese with relative GHD children treated with GH vs control group for 6 months

Variable	rhGH group (*n* = 23)	control group (*n* = 20)	*P* value
BMI SDS	2.32(0.85)	2.80(0.61)	0.041*
IGF-1(ng/ml)	702.91(246.03)	348.3(131.93)	< 0.001*
TC(mmol/L)	4.14(0.73)	4.29(0.99)	0.584
HDL-C (mmol/L)	1.45(0.40)	1.19(0.23)	0.016*#
LDL-C (mmol/L)	2.20(0.45)	2.63(0.76)	0.027*
TG(mmol/L)	1.02(0.38)	1.18(0.45)	0.261#
ALT(U/L)	16.70(6.72)	45.20(46.62)	0.002*#
AST(U/L)	21.26(5.72)	32.3(17.68)	0.006*#
Insulin(uIU/mL)	27.06(10.99)	23.26(9.78)	0.241
FBG(mmol/L)	5.46(0.41)	5.25(0.48)	0.123
HOMA-IR	6.62(2.86)	5.44(2.38)	0.152
HbA1c (%)	5.61(0.34)	5.64(0.30)	0.79

*BMI SDS* body mass index standard deviation scores, *IGF-1* Insulin-like growth factor 1, *TC* total cholesterol, *HDL-C* High density lipoprotein cholesterol, *LDL-C* Low density lipoprotein cholesterol, *TG* triglycerides, *ALT* alanine aminotransferase, *AST* aspartate aminotransferase, *FBG* fasting blood glucose, *HOMA-IR* homeostasis model assessment of IR, *HbA1c* Glycosylated haemoglobin

*P<0.05

#*P* value reported for log transformed values, but values in the table represent a back transformation to the original

#### Effects of rhGH administration on BMI SDS

**Fig. 2:**
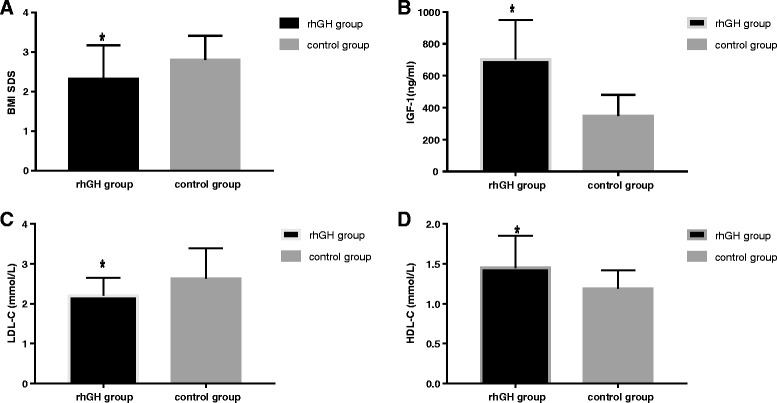
After 6 months, change in BMI SDS, IGF-1, LDL-C and HDL-C in rhGH treatment group compared with untreated control group. * *P*<0.05 vs control group

The BMI SDS in the rhGH treatment group and control group at baseline were 2.64 ± 0.74 and 2.73 ± 0.71 (*P* = 0.676), respectively (Table 1). At the 3-month visit, there was no significant difference in the BMI SDS between the rhGH treatment and the control group (2.42 ± 0.77 vs. 2.68 ± 0.53; *P* = 0.211) (Table 2). At the 6-month visit, the BMI SDS of subjects in the rhGH treatment group were significantly reduced compared with controls (2.32 ± 0.85 vs. 2.80 ± 0.61; *P* = 0.041) (Table 3, Fig. 2a). This reduction remained significant after controlling for baseline age, gender and IGF-1 level (*P* = 0.035).

#### Effects of rhGH administration on IGF-1 level

IGF-1 in the rhGH treatment group has no significant difference compared with those subjects in the control group at baseline (241.47 ± 105.98 vs. 291.10 ± 151.95 ng/mL, *P* = 0.321) (Table 1). At the 3-month visit, there was a significant increase in IGF-1 in the rhGH treatment group compared with the control group (587.35 ± 271.74 vs. 345.60 ± 131.75 ng/mL, *P* = 0.001) (Table 2). At the 6-month visit, IGF-1 level increased during rhGH treatment, in comparison with the control group (702.91 ± 246.03 vs. 348.3 ± 131.93 ng/mL, *P* < 0.001) (Table 3, Fig. 2b). The increase remained significant after controlling for baseline age, gender (*P* < 0.001).

#### Effects of rhGH administration on lipid profiles and live enzyme

At baseline, TC, HDL-C, LDL-C, TG of the rhGH treatment group did not differ from the control groups. At the 3-month visit, HDL-C increased significantly during rhGH treatment, in comparison with the control group (1.45 ± 0.38 vs. 1.17 ± 0.24 mmol/L, *P* = 0.005), whereas TC, LDL-C and TG have no significant difference between the rhGH treatment and the control group (Table 2). At the 6-month visit, LDL-C values decreased (2.20 ± 0.45 vs. 2.63 ± 0.76 mmol/L, *P* = 0.027) (Fig. 2c) and HDL-C level increased (1.45 ± 0.40 vs. 1.19 ± 0.23 mmol/L, *P* = 0.016) (Fig. 2d) in the rhGH treatment group compared with the control group. There were no significant differences in TC and TG in two groups (Table 3). Furthermore, results from multivariate regression analysis showed that LDL-C values remain decreased and HDL-C level increased in the rhGH treatment group after controlling for baseline age, gender and IGF-1 level (*P* = 0.023, *P* = 0.023).

The rhGH treatment and control groups did not differ significantly in AST and ALT at baseline. At the 3-month visit, there were no significant differences in change in AST and ALT between the rhGH treatment and the control group (Table 2). At the 6-month visit, the 6-month rhGH treatment reduced serum levels of AST (21.26 ± 5.72 vs. 32.30 ± 17.68 mmol/L, *P* = 0.006) and ALT (16.70 ± 6.72 vs. 45.20 ± 46.62 mmol/L, *P* = 0.002) compared with the control group (Table 3). The reduction remained significant after controlling for baseline age, gender and IGF-1 level (*P* = 0.007, *P* = 0.006).

#### Effects of GH administration on glucose metabolism

At baseline, there was no significant difference in insulin, FBG, HOMA-IR, HbA1c between the groups. No significant effects were elicited by rhGH treatment on insulin, FBG, HOMA-IR, HbA1c. At the 3-month visit, in rhGH treatment group, insulin, FBG, HOMA-IR increased non-significantly compared with the control group (Table 2). No difference was noted between the groups for 6-month changes in insulin, FBG, HOMA-IR and HbA1c (Table 3).

### Adverse events

There were no serious adverse events during the experiment. RhGH was well tolerated and no subject required a dose reduction. There were 4 cases of hypothyroidism during rhGH treatment, but their thyroid function returned to normal after levothyroxine treatment. Two children were found to have arthralgias, and no other adverse reactions have been observed.

## Discussion

In the study, we conducted a set of experiments based on samples of obese children with relative GHD, we found that after 6 months of rhGH treatment compared with the untreated control group, there was a significant reduction in BMI SDS. In addition, the IGF-1 levels increased in rhGH treatment group, followed by the decrease of the liver enzyme and the improvements of the lipid profile, without significant effects on increased insulin resistance and negative effects on the glucose homeostasis.

There is ample evidence that rhGH treatment can improve the body composition of obese individuals [19]. At present, most clinical trials consider rhGH treatment of obese individual weight reduction effect is limited, but in terms of reducing visceral fat, increased lean body weight may be more significant [20]. A Meta analysis in adult obesity also showed that rhGH treatment did not lead to significant weight loss in the obese subjects, but it is possible that rhGH therapy may lead to a decrease in BMI in younger subjects [21]. Since the experimental conditions are limited, we do not analyze the change of body composition. Our study showed that after 6 months of rhGH treatment, compared with controls, obese patients with relative GHD, BMI SDS decreased. This result was also consistent with the previous study.

GH lipolysis is well documented, particularly in abdominal fat [22]. Adipose tissue was recognized as a major target of GH action [23]. There is accumulating evidence that GHD of adults is associated with the increased visceral body fat and disturbed lipid metabolism. The administration of rhGH can improve lipoprotein metabolism by decreasing LDL-C and increasing HDL-C after 6 months of treatment [24]. Similar lipid profiles in adult GHD can be observed in obese children, including high levels of LDL-C, TG, as well as low levels of HDL-C. Our study demonstrated a decrease in LDL-C, and an increase in HDL-C during 6 month rhGH treatment, in comparison with the untreated control group. These results are concordant with prior studies focusing on the effects of GH in adult GHD [24] and in obese adults [21]. In addition, it is important to note that, in this study we did not observe significant change in serum TG level after rhGH treatment, and whether the result caused by the relatively short treatment time remains unknown. Thus further studies are needed to confirm.

Previous studies focusing on the effect of GH on glucose metabolism always reported the conflicting results. In adult GHD, rhGH treatment still raises glucose and insulin levels, even with lower doses [25]. Since obesity is closely associated with an increase in the prevalence of hyperinsulinemia and hyperglycemia. Therefore, whether the rhGH treatment could induce insulin resistance and increase the risk of diabetes mellitus in obese children with relative GHD is also the focus of this study. Notably, previous studies have shown that GH induced insulin resistance is rapidly increasing, but has a short duration and is rapidly reversed [26]. In 7 prepubertal boys with obesity, fasting serum insulin level was slightly increased after 3 weeks of rhGH treatment, but decreased after 3 months of rhGH treatment, and there were no significant differences in serum insulin before and after 3 months of rhGH therapy [27]. In addition, it has been confirmed that the long-term rhGH treatment can improve the insulin sensitivity in adult obesity by normalizing lipid metabolism and body composition [12, 14]. In present study, no significant effects of GH treatment on insulin, FBG, HOMA-IR and HbA1c were observed after 6 month of rhGH therapy in comparison with the untreated control group. During 6 months of treatment, GH administration has no eliciting negative effects on the glucose homeostasis. But since that the study lasted only 6 months, the effects of rhGH therapy on glucose homeostasis in obesity require further evaluation in future studies.

Prior studies suggested that the rhGH therapy could improve NAFLD and reverse histological features of nonalcoholic steatohepatitis in adults [28–31]. In this study, after 6 months of rhGH treatment, the serum ALT and AST were significantly decreased compared with the untreated control group. The improvement of liver enzyme after rhGH treatment also partly verified the therapeutic effect of rhGH treatment on NAFLD.

The innovation of our study was confirmed the effect of rhGH treatment on cardiovascular metabolic complications in obese children with relative GHD. Previous studies focusing on the effects of rhGH mostly concentrated in obese adults, or children with GHD or Prader-willi syndrome. Only two studies using rhGH in the treatment of simple obese children [27, 32]. Moreover, no existing studies did GH stimulation test to determine the status of GH secretion in obese children. Consequently, this should be one of the first study to examine the effect of rhGH in obese children with relative GHD, and thus it should have a certain scientific and clinical significance.

Limitations of the study are as follows: firstly, in this study we did not perform randomized, placebo-controlled trial, but permited to parents choose to be involved in rhGH treatment group or untreated control group. It should be noted that the design of this experiment is due to the specialty of children as all the participants and the national conditions of China. Secondly, this study did not evaluate cIMT and inflammatory markers in an observation period of 6 months. Finally, the sample size is another limitation of this study. Furture study can consider using a larger smaple size and provide more comprehensive evidences relating to our research questions.

## Conclusion

In conclusion, rhGH treatment for 6 months of obese children with relative GHD reduces BMI SDS and stabilizes IGF-1 levels, and also exerts beneficial effects on blood lipid profiles and live enzyme compared with untreated control group. Moreover, GH administration has no significant effects on increased insulin resistance and has no adverse effect on glucose homeostasis. No other serious adverse reactions occurred during the experiment.

The results of this study can provide clinical evidences for whether rhGH can be treated as a potential treatment for obese children with relative GHD. Further studies are necessary to confirm these findings, and whether long-term GH treatment is an effective strategy to avoid complications of childhood obesity needs further investigations.

## Abbreviations

ACTH: Adrenal corticotropic hormone
ALT: Alanine aminotransferase
AST: Aspartate aminotransferase
BA: Bone age
BA–CA: Bone age –chronological age
BMI: Body mass index
cIMT: Carotid intima-media thickness
COR: Cortisol
FBG: Fasting blood glucose
FT3: Free triiodothyronine
FT4: Free thyroxine
GH: Growth hormone
GHD: Growth hormone deficiency
HbA1c: Glycosylated haemoglobin
HDL-C: High density lipoprotein cholesterol
HOMA-IR: Homeostasis model assessment of IR
IGF-1: Insulin-like growth factor 1
LDL-C: Low density lipoprotein cholesterol
MRI: Magnetic resonance imaging
OGTT: Oral glucose tolerance test
rhGH: Recombinant human growth hormone
SDS: Standard deviation scores
TC: Total cholesterol
TG: Triglycerides
TSH: Thyroid-stimulating hormone
